# Characterization and Prebiotic Potential of Longan Juice Obtained by Enzymatic Conversion of Constituent Sucrose into Fructo-Oligosaccharides

**DOI:** 10.3390/molecules23102596

**Published:** 2018-10-10

**Authors:** Yongxia Cheng, Haibo Lan, Lei Zhao, Kai Wang, Zhuoyan Hu

**Affiliations:** College of Food Science, South China Agricultural University, 483, Wushan Road, Guangzhou 510642, China; chengyongxia@stu.scau.edu.cn (Y.C.); lanhaibo@stu.scau.edu.cn (H.L.); scauzl@scau.edu.cn (L.Z.)

**Keywords:** Longan juice, enzymatic conversion, oligosaccharides, bioactivity, natural products

## Abstract

The prebiotic potential of longan juice obtained by a commercial Viscozyme L for conversion of constituent sucrose to fructo-oligosaccharide was investigated. The physicochemical properties and carbohydrate composition of the longan juice was evaluated before and after enzymatic treatment. The stimulation effects of the treated longan juice on probiotic bacteria growth were also studied in vitro. The results showed that total soluble solids, yield and clarity of longan juice were all significantly improved after enzyme treatment. The water-soluble polysaccharide content, including pectin, was significantly increased. Compared with the natural longan pulp, the enzyme treated juice showed a significant decrease in sucrose content. Substantial fructo-oligosaccharides including 1-kestose and nystose were synthesized after enzyme treatment. The molecular weight distribution and the monosaccharide composition of the water-soluble polysaccharide were significantly changed by enzyme treatment. The treated longan juice and its ethanol-soluble sugar fraction promoted the growth of *Streptococus thermophiles*, *Lactobacillus acidophilus* and *Lactobacillus delbrueckii*, showing a good potential of the treated longan juice for producing functional foods and nutraceuticals.

## 1. Introduction

Longan (*Dimocarpus longan* Lour.) is an important economic fruit in southern China [1]. It has been reported that longan is rich in phenolic compounds and polysaccharides, which have excellent radical-scavenging activities, potent immune-modulatory, great antitumor effects and regulating intestinal flora effects [2,3,4]. Therefore, it is preferred not only for its desirable flavor but also for its nutritional value [5,6]. The percentage yield of hot water extract in dried longan pulp was 40.36%, the total phenol and polysaccharide content in the extract was 12.15 mg/g and 58.4 mg/g, respectively [7].

In juice processing, enzymes are commonly added during maceration, prior to the pressing of fruits to obtain satisfactory juice yield [8]. These enzymes, including cellulase, glucanases and pectinases etc., are normally able to catalyze the degradation of cellulose and pectin in cell walls [9], leading to improvements in juice clarity, juice extraction yield and in the release of bioactive compounds trapped in plant cells [10]. At the same time, physicochemical properties such as viscosity and the carbohydrate composition of juice are also impacted [11].

The main sugar compositions of longan are sucrose, glucose and fructose [12], among which sucrose is the most abundant one, accounting for about 60–70% of the total sugar content. High sucrose intake results in a high blood glucose level, which could lead to several metabolic syndromes, such as obesity, cardiovascular diseases and type II diabetes [13]. Hence, reducing sucrose content in juice is beneficial in keeping blood glucose level of consumers stable, and in preventing these metabolic syndromes [14]. One approach is to convert sucrose into fructo-oligosaccharides (FOSs) through the action of extracellular/intracellular fructosyltransferases (FTases, EC 2.4.1.9) or β-fructofuranosidases (FFases, EC 3.2.1.26) [15,16,17], or into gluco-oligosaccharides (GOSs) by glucansucrases [18]. As prebiotics, consumption of FOSs and GOSs, as well as other oligosaccharides, could modulate gut microbiota [19,20], help to create a healthy intestinal ecology [21] and improve mineral absorption in the colon [22]. Therefore, it could reduce the risk of multiple diseases, including obesity, metabolic dysfunction and immune system disorders [23]. There have been studies on the conversion of intrinsic sucrose in concentrated fruit juices into oligosaccharides through enzymatic treatments [14,24]. In these studies, finished fruit juices were used as raw materials, and sucrose conversion was an extra procedure in addition to juice processing, leading to increases in cost and time consumption of juice production. In comparison, using fresh fruit pulp as the starting material, and converting sucrose to oligosaccharides in the processing of fruit pressing would be a more economic and time saving approach. However, there has rarely been studies on this topic.

The current study uses a commercial enzyme, Viscozyme L from *Aspergillus aculeatus*, containing a mixture of cellulases, glucanases, pectinases and FFases, in longan juice pressing, and aims to convert sucrose to FOSs in longan pulp through transfructosylation by FFases, on the basis of achieving improved juice extraction yield and clarity by cellulases, glucanases and pectinases [25]. Physicochemical properties of the enzyme treated longan juices including total soluble solids, yield, clarity and color were evaluated, and changes in sugar composition were also evaluated. In addition, the effects of longan juices, its ethanol-soluble sugar fractions and water-soluble polysaccharide fractions on the promotion of lactobacilli were also studied in vitro. The results of this study will provide theoretic and practical guidelines for producing prebiotic longan juices.

## 2. Results and Discussion

In the present work, longan pulp was treated by Viscozyme L during juice production, and the results showed that enzyme treatment not only improved the processing characteristics of longan juice including yield, clarity and total soluble solids, but also changed its carbohydrate compositions. Two fructo-oligosaccharides, 1-kestose and nystose, were observed after enzyme treatment along with significant decreases in sucrose content, indicating the conversion of sucrose to 1-kestose and nystose by the enzymes. The treated longan juice and its ethanol-soluble sugar fraction also stimulated the growth of *Lactobacillus* strains including strain *S. thermophiles*, *L. acidophilus* and *L. delbrueckii*.

### 2.1. Physical Properties of Longan Juice

The physical properties of the enzymatic treated longan juice samples (2 and 5 h) were compared with the untreated longan juice (0 h), and the results are presented in Table 1.

The juice yield and total solid content (76.91% and 19.33 °Brix, respectively) were both significantly improved after enzyme treatment for 2 h, and they were further increased to 93.03% and 20.13 °Brix, respectively, after enzyme treatment for 5 h. A similar trend was also observed in the clarity of longan juice. The freshly pressed longan juice was relatively turbid and cloudy in appearance, with a clarity of 45.80%. This is probably due to colloidal dispersion of polysaccharides such as pectin, cellulose, and hemicellulose components present in the forms of disrupted cell wall and cell materials [26]. These polysaccharides could also lead to low juice yield [8]. After enzymatic treatment for 2 h, the clarity of the longan juice was apparently improved to 66.27%. And when the enzymatic treatment was extended to 5 h, its clarity was further increased to 82.07%. This could be explained by the good activities of the enzymes. The commercial enzyme, Viscozyme L, used in this study consists of a complex of cellulolytic and pectinolytic enzymes that act in different ways to catalyze the hydrolysis of cellulosic and pectic substances in fruits [8]. These enzymes could effectively break down cellulosic and pectic substances in longan juice, leading to significant increases in juice clarity, juice yield and total solid content.

Enzyme treatment also significantly changed the color of longan juice (Table 1). The results showed that the lightness L* value of freshly prepared longan juice was significantly reduced from 52.81 to 47.65 after enzymatic treatment for 5 h. In contrast, the redness value a* and yellowness value b* were significantly increased after enzymatic treatment. This is probably because of the browning effect (including enzymatic browning and non-enzymatic browning) happened under the enzyme treatment conditions (pH 6 and 55 °C).

### 2.2. Chemical Composition of Longan Juice

The chemical compositions of longan juice before and after enzymatic treatment are presented in Table 2.

The total sugar in longan juice was studied in three groups: ethanol-soluble sugars, water-soluble polysaccharides and insoluble fiber. The results showed that the ethanol-soluble sugar content was not significantly changed after enzymatic treatment, with the amount ranging from 175.78 to 175.92 mg/g. The amount of water-soluble polysaccharides increased dramatically from 3.61 to 8.30 mg/g after enzymatic treatment for 5 h, whereas that of insoluble fiber decreased significantly with the enzymatic treatment time extended. This is because, during the enzymatic treatment, a significant quantity of insoluble fiber could be hydrolyzed to oligomers, which were recovered in the group of water-soluble polysaccharides. This is in agreement with results for enzymatic conversion of date fruit fiber reported by Mrabet et al. (2017) [27]. The water-soluble pectin content of freshly prepared longan juice was 0.07 mg/g, and it was significantly increased after enzyme treatment, reaching 0.20 mg/g after 5 h of treatment. During enzyme treatment, the enzymes could degrade cell walls, leading to the release of pectin from the cell wall matrix. These enzymes could also partially depolymerize and solubilize pectin [28], resulting in an increase in the amount of water-soluble pectin.

The total polyphenol compounds and total soluble protein concentrations were decreased along with the enzymatic hydrolysis, with the amounts decreased from 12.23 µg/g and 3.06 mg/g to 8.69 µg/g and 0.93 mg/g, respectively. The reduction in polyphenols and soluble protein content in this study might because under the treatment temperature condition (55 °C), polyphenols was partially oxidized and proteins was denatured.

### 2.3. Ethanol-Soluble Sugars Profile in Longan Juice

**Figure 1:**
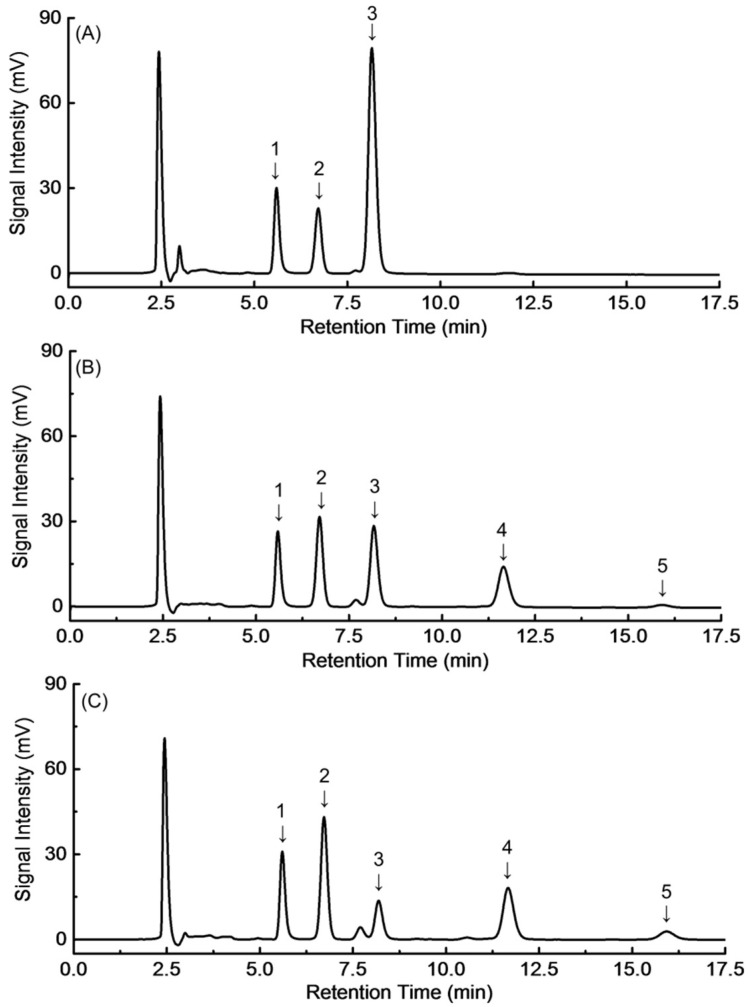
Representative high performance liquid chromatography (HPLC) profiles of fresh longan juice (**A**) and enzyme treated longan juices for 2 h (**B**), and 5 h (**C**). Identified peaks are as follows: 1, fructose; 2, glucose; 3, sucrose; 4, 1-kestose; 5, nystose.

Figure 1 shows the monosaccharide and oligosaccharide profiles of enzyme treated longan juice as compared with untreated longan juice. Under the chromatographic condition employed, the major carbohydrates detected in the untreated longan juice were fructose, glucose and sucrose, eluted as three single peaks with retention times at approximately 5.6, 6.7 and 8.1 min, respectively (Figure 1A).

Figure 1B shows the HPLC profile of longan juice after enzyme treatment for 2 h. In addition to the unidentified small peak eluted at 7.6 min, the chromatogram showed two additional peaks with retention times at 11.6 min and 15.8 min, indicating the production of 1-kestose and nystose. At the same time, the sucrose content was significantly reduced with the increase in enzyme treatment time (Table 3). These peaks of 1-kestose and nystose are not observed in the HPLC profile of untreated longan juice, meaning that these carbohydrates are not present naturally in longan. Instead they are most likely to be converted from intrinsic sucrose during enzyme treatment. Similar peaks were observed in longan juice after 5 h enzymatic treatment (Figure 1C), showing the same monosaccharide and oligosaccharide components.

The content of each sugar component was calculated using the linear equation between peak area and concentration of each standard, and the results are given in Table 3.

It was observed in the results that the sucrose content was dramatically reduced from 110.98 mg/g to 42.25 mg/g after 2 h of enzymatic treatment, and it was further decreased to 17.11 mg/g after 5 h of treatment. At the same time, significant amounts of 1-kestose (42.25 mg/g) and nystose (7.16 mg/g) were newly produced after enzymatic treatment for 2 h, and larger amounts (47.52 and 14.08 mg/g respectively) were produced after 5 h of enzyme treatment. During this process, the fructose content was slightly changed, while glucose content was significantly increased. These changes could be attributed to the activity of FFases in Viscozyme L. It has been reported that FFases have transfructosylation activity in sucrose solutions [15]. FFases could disconnect the β-2,1 glycosidic bonds between glucose and fructose units, and then produce FOS by transferring the fructosyl to another sucrose [29]. The prebiotic longan juices obtained in the present work are low caloric because a low amount of residual sucrose were presented after the enzyme treatment due to the fructose incorporation into the oligosaccharides chain. The enzymatic conversion decreased the original sucrose content at least seven-fold.

### 2.4. Molecular Size Distribution of Water-Soluble Polysaccharides

**Figure 2:**
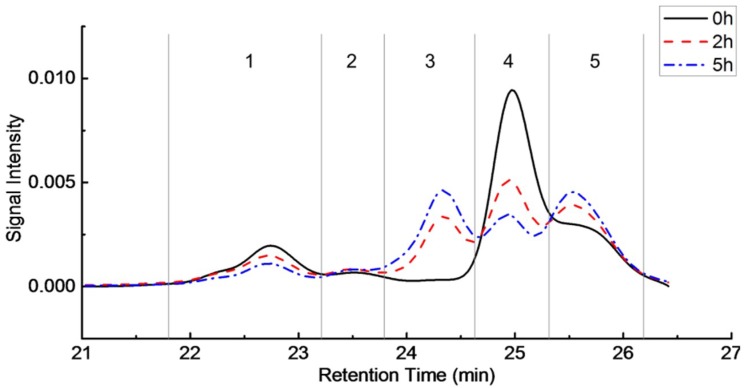
Molecular size distribution of water-soluble polysaccharides in enzyme treated longan juice for 2 h and 5 h, as compared with untreated longan juice (0 h).

The molecular size distribution of water-soluble polysaccharides in longan juice before (0 h) and after enzyme treatment for 2 and 5 h were characterized by Gel Permeation Chromatography (GPC), and the results are shown in Figure 2. All distributions were normalized to achieve the same area under each curve. Therefore, impacts of differences in sample concentration on the distribution curve were eliminated. Five peaks were observed in the molecular size distribution of all the three samples, with peak maximums exhibited at 22.7, 23.5, 24.3, 24.9 and 25.5 min respectively, meaning five fractions present in each sample. Area under each peak was calculated to represent the relative proportion of each fraction, with results exhibiting in Table 4.

In the untreated longan juice sample, fraction 4 is the most abundant, followed by fraction 5 and fraction 1. The amounts of fractions 2 and 3 were the lowest. Significant changes were observed in the molecular size distribution of the water soluble polysaccharides in longan juice after enzymatic treatment, meaning that Viscozyme L could alter the polysaccharide profile of longan juice, and this alternation extent is hydrolysis time dependent. After enzyme treatment, the heights of peak 1 and peak 4 were apparently decreased, whereas those of peak 3 and peak 5 were increased. As peak 2 is quite small in comparison with other peaks, change in its peak height is not apparent, although a significant decrease was observed in the sample after 5 h of enzymatic treatment (Table 2). These could be because that after enzyme treatment, fraction 1, representing polysaccharides with the largest sizes, were hydrolyzed to smaller ones, resulting in increases in the proportions of fraction 3 and fraction 5. Similarly, polysaccharides in fraction 4 could also be hydrolyzed to smaller ones during hydrolysis by Viscozyme L, leading to increases in the proportion of fraction 5. Similar results were reported by multiple researchers, showing that macromolecule polysaccharides could be hydrolyzed into small molecules by enzymatic treatment [27]. The increased proportions of fractions 3 and 5 could also be partially attributed to intracellular polysaccharides released by the decomposition of plant cell wall after enzyme treatment [30].

### 2.5. Monosaccharide and Uronic Acid Composition of Water-Soluble Polysaccharide and Insoluble Fiber

**Figure 3:**
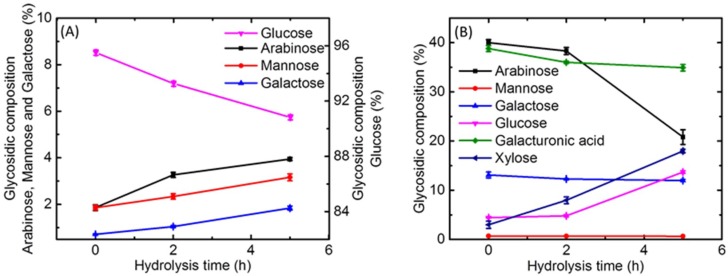
Glycosidic composition and corresponding molar percentages during the hydrolysis developing from 0 h to 5 h. (**A**), the water-soluble polysaccharide; (**B**), the insoluble fiber.

The monosaccharide and uronic acid composition of water-soluble polysaccharide and insoluble fiber fractions of longan juices were analyzed using high performance anion-exchange chromatography (HPAEC), and the results are shown in Figure 3. The values are expressed as relative proportions.

As shown in Figure 3A, the water-soluble polysaccharide fraction in untreated longan juice mainly consisted of glucose (95.50%), arabinose (1.86%), mannose (1.86%) and galactose (0.71%), among which glucose was the most abundant. The proportions of arabinose, mannose and galactose were significantly increased after enzyme treatment for 2 h, and their proportions were further increased, reaching 3.95%, 3.16% and 1.84% after 5 h of enzyme treatment. In contrast, the proportion of glucose reduced with increased enzyme treatment time length, dropping to 90.82% after 5 h of enzyme treatment. These results show that more arabinose, mannose and galactose in the water soluble polysaccharide fraction were produced during enzyme treatment of longan juice, whereas glucose had less production than others, leading to a decline in its relative proportions.

In addition to glucose, arabinose, maltose and galactose, mannose and galacturonic acid were also detected in the insoluble fiber fractions (Figure 3B). Among them, arabinose and galacturonic acid were the main components before enzyme treatment, with the proportions being 40.00% and 38.81% respectively. The proportion of arabinose slightly decreased after 2 h of enzymatic treatment of longan juice, and it was remarkably reduced to 20.81% after 5 h of treatment. Similarly, gradual reductions were also observed in the proportions of galacturonic acid and galactose after enzyme treatment for 2 h and 5 h. In comparison, significant and gradual increases were present in the proportions of xylose and glucose after enzyme treatment of longan juice for 2 h, and a dramatic increase was observed after 5 h of enzyme treatment (reaching 17.97 and 13.70%, respectively). Viscozyme L has potent activities of arabinose hydrolase and pectinase [27], which were usually employed to degrade cell walls so as to improve juice yield and the release of the water soluble polysaccharide. This is an important reason for the significant decreases in the proportions of arabinose and galacturonic acid in the insoluble fiber.

### 2.6. The Growth Effect of Treated Longan Juice on Probiotic Bacteria

**Figure 4:**
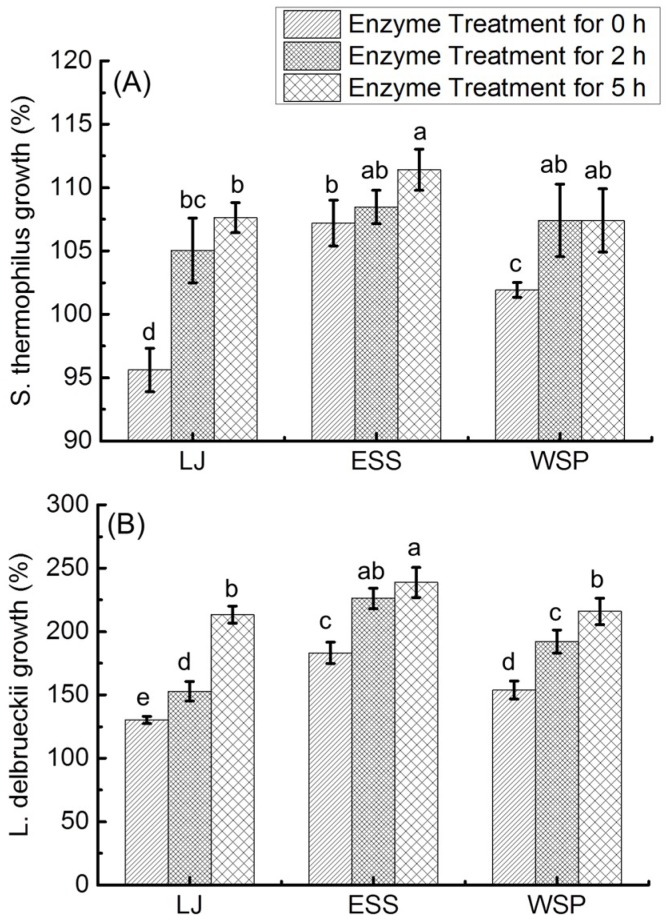

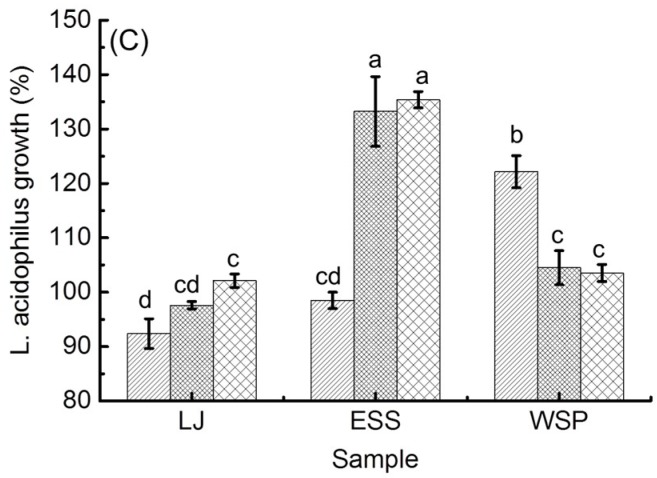
Influences of enzyme treated longan juices (LJ), their ethanol-soluble sugar (ESS) and water-soluble polysaccharide (WSP) components in longan juices after enzyme treatment for 0 h, 2 h and 5 h on the growth stimulation activity of (**A**) *S. thermophiles* (%), (**B**) *L. delbrueckii* (%) and (**C**) *L. acidophilus* (%). The values were calculated on the basis of glucose activity (taken as 100%). Different letters on the bars indicate significantly different (*p* < 0.05).

The enzyme treated longan juices (LJ) (enzyme treated for 0, 2 and 5 h), their ethanol-soluble sugar (ESS) fraction and water-soluble polysaccharide (WSP) fraction were used to study their impacts on the stimulation of *lactobacilli in vitro*, and the results are shown in Figure 4. After 48 h of incubation, the maximum growth of *L. delbrueckii*, *S. thermophiles* and *L. acidophilus* was observed. The growth rate values obtained at 48 h were accepted for further statistical analyses, respectively.

Figure 4A presents the influences of the samples on the growth of reference strain *S. thermophiles*. Growth rates of *S. thermophiles* stimulated by LJ-0h, ESS-0h and WSP-0h were 95.61%, 107.19% and 101.92% respectively. In comparison to their untreated longan juice counterparts, the growth rates of LJ and WSP after enzyme treatment for 2 and 5 h were significantly increased. No significant differences were observed in the ESS fractions from longan juice before (ESS-0h) and after enzyme treatment for 2 h (ESS-2h). However, the ESS fraction of longan juice underneath enzyme treatment for 5 h (ESS-5h) significantly promoted the growth of *S. thermophiles* compared with ESS from the untreated longan juice. These results show that enzymatic treated longan juice as well as its ESS and WSP fractions could accelerate the growth of *S. thermophiles*.

As seen from Figure 4B, the growth rates of *L. delbrueckii* stimulated by the 9 samples had similar trends to *S. thermophiles* (results shown in Figure 4A). After enzyme treatment, longan juice and its ESS and WSP fractions were more beneficial to the growth of stain *L. delbrueckii*, and the improvement effect was increased to a higher extent with the extension in the enzyme treatment time length. These results indicate that components produced during enzymatic treatment of longan juice are favored by strain *L. delbrueckii*, and these components include both FOS in the ESS and WSP.

The impacts of different samples on the growth of *L. acidophilus* are presented in Figure 4C. Before enzyme treatment, longan juice and its ESS fraction did not increase the growth of *L. acidophilus*, as compared with glucose, whereas the growth rate was promoted to 122.14% by the WSP fraction from untreated longan juice. After enzymatic treatment of longan juice for over 2 h, its ESS fraction further increased the growth rate of *L. acidophilus* to around 133.23 and 135.39%. In contrast, the acceleration effect of WSP from enzyme treated longan juice (104.48 and 103.53%) was not as remarkable as its untreated longan juice counterpart (122.14%). These results show that components produced during enzyme treatment of longan juice could improve the growth of *L. acidophilus*, and that these effective components are most possibly the FOS in the ESS fraction produced during enzyme treatment. This is in good agreement with previous studies showing that FOS supports the growth of *L. acidophilus* [31].

Polysaccharides and FOS in longan juice could promote the growth of probiotics and might have the effect of regulating intestinal flora. The intestinal microbial flora might play a crucial role in the development of metabolic syndrome and associated disorders [32]. Therefore, longan juice might have the effect of regulating metabolic syndrome, but no literature reports are available in this area. It is necessary to study the effect of longan juice on chronic diseases, such as obesity, cardiovascular disease or diabetes.

## 3. Materials and Methods

### 3.1. Materials

Longan (*Dimocarpus longan* Lour. cultivars ‘Chuliang’) fruit was obtained from a local commercial orchard in Gaozhou, Guangdong province, China. Fruits without visible damage and diseases were manually harvested at maturity stage in August 2016. Viscozyme L from *Aspergillus aculeatus*, purchased from Novo Nordisk Ferment Ltd. (Dittingen, Switzerland), was used for the enzymatic treatment of longan pulp. 1-kestose, nystose, sucrose, glucose and fructose standards were purchased from Sigma-Aldrich Pty. Ltd. (St. Louis, MO, USA). *Streptococus thermophilus* CICC 6223, *Lactobacillus acidophilus* ATCC 4356 and *Lactobacillus delbrueckii* CICC 6045 were purchased from Microbial Culture Collection Center of Guangdong Institute of Microbiology (GIMCC, Guangzhou, China). Other reagents were purchased from Merck (Darmstadt, Germany) or commercial sources in Guangzhou.

### 3.2. Enzymatic Treatment of Longan Pulp Samples

Longan fruits were manually peeled and deseeded and the arils were pulped using a domestic blender and the pulp was boiled and then cooled for enzymatic treatment. Considering that the published works have stated the optimum temperature and pH for FOS-producing enzymes activity between 50 to 60 °C and 4.5 to 6.5, respectively [17], the longan pulp (natural pH 6.8) was adjusted to pH 6.0 with 1 M citric acid for enzymatic treatment. A total of 50 g of longan pulp samples were weighed into a flask and then treated with 300 mg of Viscozyme L at pH 6.0 and 55 °C for 2 h and 5 h respectively. The treated longan pulp samples were then immediately heated in a 90 °C water bath for 3 min to stop reaction [11], and centrifuged at 3000× *g* for 15 min. The supernatant (clear juice) was collected and filtered through a filter paper (Whatman^®^, Meterstone, British) using a diaphragm vacuum pump (MZ 2C, Vacuubrand, Germany) [15]. The residue was freeze-dried by a freeze dryer (FD-1C, Beijing Bo Medical Devices Co., Ltd., Beijing China) for determining insoluble fiber content. The received clear juice and freeze-dried residue samples were stored at −20 °C before analysis.

### 3.3. Total Soluble Solid Content, pH, Yield, Clarity and Color Measurement

The total soluble solid content of sample was measured using a portable refractometer (WZ103, Shanghai Science and Technology Co., Shanghai, China), and the results were expressed as °Brix.

The pH of sample was measured using a pH meter (FE320, Mettler Toledo Instruments, Zurich, Switzerland).

Longan juice yield was calculated using the following formula [33]:

(1) $$\mathrm{Yield}\left(\%\frac{v}{v}\right)=\frac{\mathrm{Volume}\;\mathrm{of}\;\mathrm{clear}\;\mathrm{fruit}\;\mathrm{juice}\;\left(\mathrm{mL}\right)}{\mathrm{Volume}\;\mathrm{of}\;\mathrm{raw}\;\mathrm{pulp}\;\left(\mathrm{mL}\right)}\times 100\%$$

Clarity was determined by measuring the absorbance at 660 nm using a UV-Vis Spectrophotometer (UVmini-1240; Shimadzu Corporation, Kyoto, Japan) with distilled water as the reference [34].

The color of clarified juice was determined using a colorimeter (Spectrophotometer CM-3500d, Minolta Co. Ltd., Osaka, Japan) according to method reported by [35]. Three color values were determined: L* (whiteness or brightness/darkness), a* (redness/greenness), and b* (yellowness/blueness).

### 3.4. Proximate Composition of the Juice

The longan juice samples, after enzymatic treatment for different time lengths (0, 2, and 5 h), were analyzed for their contents of ethanol-soluble sugars, water-soluble polysaccharide, insoluble fiber, soluble pectin, total polyphenol and soluble protein.

The content of water-soluble polysaccharides was determined using the phenol-sulfuric acid colorimetry following the method of [36]. Polysaccharides were extracted from clear longan juice using 80% ethanol, and then dissolved in deionized water. A proportion was mixed with phenol solution and sulfuric acid solution, and the absorbance was measured at 490 nm with a Shimadzu UVmini-1240 UV-Vis Spectrophotometer (Tokyo, Japan) against a blank (20 mL of deionized water plus reagents) after an incubation step at 30 °C for 20 min. Glucose was used as the standard.

The content of insoluble fiber was determined using the method of [37]. Briefly, 1 g of the dried residue sample (Section 2.2) was subjected to sequential enzymatic digestion by thermal-stable α-amylase, protease and amyloglucosidase. The residue was filtered, successively washed with warm distilled water, 78% ethanol, followed by 95% ethanol and then acetone. It was finally dried and weighed. A proportion was incinerated at 525 °C to determine ash content, and the rest was analyzed for protein following the Kjeldahl method. The content of insoluble fiber was corrected for ash and protein contents.

The content of soluble pectin in longan juice was determined using the spectrophotometry method described by [38].

The total content of polyphenol compounds in longan juice was determined following the method of [39] with modifications. Briefly, 2 mL of clear juice (Section 2.2) were mixed with 4 mL of 50% ethanol, and were kept in ice bath for 30 min. Then, 0.2 mL of the mixture was transferred into a 10 mL brown volumetric flask and 0.5 mL of Folin–Ciocalteu reagent was added and allowed to react for 5 min in dark, followed by the addition of 1.5 mL of 20% sodium carbonate solution. The absorbance of solution was measured at 765 nm, after 2 h of incubation in dark. The content of total polyphenols was expressed as µg gallic acid equivalents (GAE) in 1 g sample.

The total soluble protein concentration of longan juice was determined using the Bradford protein assay according to the method of [40]. Clear juice (1 g, prepared as described in Section 2.2) was added into 5 mL Coomassie Brilliant Blue G–250. After 20 min of incubation, the absorbance of solution was measured at 595 nm.

### 3.5. Quantitative Analysis of Ethanol-Soluble Sugars using HPLC

An HPLC system (Shimadzu series LC-20AT, Kyoto, Japan) coupled with a refractive index (RI) detector was used to evaluate mono- and oligo-saccharide contents in the enzyme treated samples. The ethanol-soluble sugar of each sample was extracted by 80% (*v*/*v*) ethanol and adjusted the concentration by deionized water. It was filtered through a Polytetrafluoroethylene (PTFE) filter (0.45 µm) before analyzed. A proportion of the prepared sample (10 μL) was injected, using HPLC grade acetonitrile/water (at a ratio of 70:30) as the mobile phase at a flow rate of 1 mL/min. It was then separated using a polymeric amino column (Shodex Asahipak NH2P-50 4E, 250 × 4.6 mm, Japan) at 30 °C, and determined using a refractive index detector at 30 °C. Standard solutions of 1-Kestose, nystose, sucrose, glucose and fructose (Sigma-Aldrich Chemical Co., St. Louis, MO, USA) were used for interpretation and quantification of sugars in the samples.

### 3.6. Evaluation of the Molecular Size Changes of Water-Soluble Polysaccharides using High-Performance Gel Permeation Chromatography (GPC)

The clear juice (prepared as described in Section 2.2) was precipitated with 4 volumes of 80% ethanol, and the precipitate was freeze-dried to prepare water-soluble polysaccharide samples. The water-soluble polysaccharide samples were redissolved in deionized water, and filtered with 0.45 µm membrane. The molecular size distribution of the water-soluble polysaccharides was analyzed using a GPC system (Shimadzu LC-20AT) equipped with a refractive index detector (Kyoto, Japan). Samples were separated using Ultrahydrogel linear (Waters, Milford, MA, USA) and G3000PWXL (Tosoh Corporation, Tokyo, Japan) columns. The elution buffer was 0.2 mol/L sodium nitrate at a flow rate of 0.4 mL/min.

### 3.7. Monosaccharide Composition of Water-Soluble Polysaccharide and Insoluble Fiber

Monosaccharide and uronic acid compositions in the water-soluble polysaccharide and insoluble fiber samples (10 mg) were determined by hydrolysis with 3 mL of 2 M H_2_SO_4_ at 100 °C for 6 h. After the hydrolysis was completed, the mixture was cooled to room temperature and neutralized with barium carbonate. The samples analysis were carried out using a high performance anion-exchange chromatography with pulsed amperometric detection (HPAEC-PAD, Dionex ICS-3000, Sunnyvale, CA, USA) equipped with a CarboPacTM PA20 guard column and a CarboPacTM PA20 analytical column (4 mm × 250 mm). Glucose, galactose, mannose, arabinose, xylose and galacturonic acid were used as standards.

### 3.8. The Growth Effect of Longan Juice on Probiotic Bacteria

The growth of lactobacilli strains (*Streptococus thermophilus* CICC 6223, *Lactobacillus acidophilus* ATCC4356 and *Lactobacillus delbrueckii* CICC 6045) in the presence of the enzyme treated longan juice, its ethanol soluble sugar fraction, and its water-soluble polysaccharide fraction was determined following the method of [41] under anaerobic conditions. The test was performed in U-shaped 96-plates and read the absorbance at 600 nm with an ELISA reader (Bio-Rad iMark, Bio-Rad Laboratories, Inc., Hercules, CA, USA). De Man-Rogosa-Sharpe (MRS) broth (200 µL) was used, in which glucose was replaced with 10% enzyme treated longan juice, 2% ethanol-soluble sugar fraction and 2% water-soluble polysaccharide fraction of enzyme treated longan juices, respectively. The amount of sugars in enzyme treated longan juice, ethanol-soluble sugar fraction and water-soluble polysaccharide fraction above were the same as the amount of glucose in the MRS broth media. It was then inoculated with 20 µL of *S. thermophiles*, *L. acidophilus* or *L. delbrueckii* (contains 150 × 10^6^ CFU/mL) respectively, each medium without inoculation corresponded to a blank, and then the plates were incubated at 37 °C for up to 72 h. The absorbance was measured after 0, 24, 48 and 72 h of incubation using an ELISA reader at a wavelength of 600 nm. The results were presented as the percentage of probiotic bacteria growth in the presence of each longan carbohydrate in comparison with the growth in the glucose-containing medium (taken as 100%).

### 3.9. Statistical Analysis

All analyses were carried out in triplicates. The mean and standard deviation were analyzed by SPSS statistical software (version 19.0, SPSS Inc., Chicago, IL, USA) using analysis of variance (ANOVA) with the general linear model and Tukey’s pairwise comparisons with the confidence level at 95%. Significant differences of the mean values were determined at *p* < 0.05.

## 4. Conclusions

Results in this study showed that enzymatic treatment of longan pulp not only improved the processing characteristics of longan juice including yield, clarity and total soluble solids, but also changed its carbohydrate compositions. The amount of insoluble fiber was decreased and the contents of pectin and water-soluble polysaccharides were increased with the enzymatic treatment, attributing to the hydrolysis of insoluble fiber to oligomers and release of pectin from degradation of cell wall matrix. Two FOSs, 1-kestose and nystose, were observed after enzyme treatment along with significant decreases in sucrose content, indicating the conversion of sucrose to 1-kestose and nystose by the enzymes.

With enzymatic treatment, the molecular size distribution of the water-soluble polysaccharides was significantly changed. The monosaccharide and uronic acid composition of water-soluble polysaccharide and insoluble fiber were also apparently altered. The stimulation of treated longan juices and ethanol-soluble sugars on the reference strain *S. thermophilus*, *L. acidophilus* and *L. delbrueckii* were all significantly improved. The results indicated that enzymatic treatment is a good way to change the juice carbohydrate compositions and to reduce the calories of longan juice. It would have a positive impact on the health juice processing industry.

## Figures and Tables

**Table 1:** Physical properties of longan juice from different enzymatic treatment time *.

Treated Time (h)	Yield (%)	Clarity (T/%)	Total Solid Content (°Brix)	Color Parameters		
				L *	A *	B *
0	76.91 ± 3.23 **^c^**	45.80 ± 2.07 **^c^**	19.33 ± 0.06 **^c^**	52.81 ± 0.17 **^a^**	8.28 ± 0.03 **^c^**	12.90 ± 0.19 **^b^**
2	85.78 ± 1.82 **^b^**	66.27 ± 2.92 **^b^**	19.87 ± 0.06 **^b^**	49.41 ± 0.39 **^b^**	8.63 ± 0.04 **^b^**	14.21 ± 0.10 **^a^**
5	93.03 ± 1.95 **^a^**	82.07 ± 1.15 **^a^**	20.13 ± 0.06 **^a^**	47.65 ± 0.09 **^c^**	9.01 ± 0.16 **^a^**	14.04 ± 0.12 **^a^**

* The data shown were mean ± standard deviation of three replicates. Values with different letters in the same column were significantly different at *p* < 0.05.

**Table 2:** Chemical composition of enzyme treated longan juice *.

Treated Time (h)	Ethanol-Soluble Sugars (mg/g)	Water-Soluble Polysaccharides (mg/g)	Insoluble Fiber (mg/g)	Water-Soluble Pectin (mg/g)	Polyphenols (µg/g)	Soluble Protein (mg/g)
0	175.78 ± 0.94 ^a^	3.61 ± 0.15 ^c^	11.74 ± 0.49 ^a^	0.07 ± 0.00 ^c^	12.23 ± 0.15 ^a^	3.06 ± 0.05 ^a^
2	175.82 ± 1.48 ^a^	4.88 ± 0.16 ^b^	8.98 ± 0.15 ^b^	0.16 ± 0.00 ^b^	10.99 ± 0.11 ^b^	1.02 ± 0.01 ^b^
5	175.92 ± 1.68 ^a^	8.30 ± 0.27 ^a^	6.07 ± 0.04 ^c^	0.20 ± 0.01 ^a^	8.69 ± 0.29 ^c^	0.93 ± 0.01 ^c^

* The data shown were mean ± standard deviation of three replicates. Values with different letters in the same column were significantly different at *p* < 0.05.

**Table 3:** Sugar contents of longan juice before and after enzymatic treatment *.

Treated Time (h)	Fructose (mg/g)	Glucose (mg/g)	Sucrose (mg/g)	1-Kestose (mg/g)	Nystose (mg/g)	FOSs (mg/g)
0	27.92 ± 0.08 ^a^	36.87 ± 0.06 ^c^	110.98 ± 0.80 ^a^	0	0	0
2	27.27 ± 0.20 ^b^	56.89 ± 0.51 ^b^	42.25 ± 0.38 ^b^	42.25 ± 0.68 ^b^	7.16 ± 0.18 ^b^	49.40 ± 0.58 ^b^
5	28.17 ± 0.23 ^a^	69.05 ± 0.30 ^a^	17.11 ± 0.26 ^c^	47.52 ± 0.51 ^a^	14.08 ± 0.45 ^a^	61.59 ± 0.93 ^a^

* The data shown were mean ± standard deviation of three replicates. Values with different letters in the same column were significantly different at *p* < 0.05.

**Table 4:** Relative proportions of the five fractions of the water-soluble polysaccharides in longan juice before (0 h) and after enzyme treatment for 2 h and 5 h *.

Treated Time (h)	Fraction 1 (%)	Fraction 2 (%)	Fraction 3 (%)	Fraction 4 (%)	Fraction 5 (%)
0	17.67 ± 0.61 ^a^	4.87 ± 0.41 ^a^	2.22 ± 0.04 ^c^	55.12 ± 2.12 ^a^	20.13 ± 1.05 ^c^
2	13.91 ± 0.46 ^b^	4.64 ± 0.27 ^a^	20.32 ± 0.28 ^b^	30.05 ± 0.05 ^b^	31.10 ± 0.14 ^b^
5	9.56 ± 0.13 ^c^	4.11 ± 0.07 ^b^	31.47 ± 0.20 ^a^	18.33 ± 0.36 ^c^	36.43 ± 0.04 ^a^

* Values with different letters in the same column were significantly different at *p* < 0.05.
